# An Integrated Model Based on a Six-Gene Signature Predicts Overall Survival in Patients With Hepatocellular Carcinoma

**DOI:** 10.3389/fgene.2019.01323

**Published:** 2020-01-14

**Authors:** Wenli Li, Jianjun Lu, Zhanzhong Ma, Jiafeng Zhao, Jun Liu

**Affiliations:** ^1^ Department of Clinical Laboratory, Yue Bei People's Hospital, Shantou University Medical College, Shaoguan, China; ^2^ Department of Reproductive Medicine Center, The Affiliated Yue Bei People's Hospital of Shantou University Medical College, Shaoguan, China; ^3^ The Second School of Clinical Medicine, Southern Medical University, Guangzhou, China; ^4^ Department of Medical Services, First Affiliated Hospital of Sun Yat-sen University, Guangzhou, China; ^5^ Morning Star Academic Cooperation, Shanghai, China; ^6^ Department of Hepatobiliary Surgery, Yue Bei People's Hospital, Shantou University Medical College, Shaoguan, China

**Keywords:** hepatocellular carcinoma, overall survival, risk score, mRNA signature, weighted gene co-expression network analysis

## Abstract

**Background:** Nowadays, clinical treatment outcomes of patients with hepatocellular carcinoma (HCC) have been improved. However, due to the complexity of the molecular mechanisms, the recurrence rate and mortality in HCC inpatients are still at a high level. Therefore, there is an urgent need in screening biomarkers of HCC to show therapeutic effects and improve the prognosis.

**Methods:** In this study, we aim to establish a gene signature that can predict the prognosis of HCC patients by downloading and analyzing RNA sequencing data and clinical information from three independent public databases. Firstly, we applied the limma R package to analyze biomarkers by the genetic data and clinical information downloaded from the Gene Expression Omnibus database (GEO), and then used the least absolute shrinkage and selection operator (LASSO) Cox regression and survival analysis to establish a gene signature and a prediction model by data from the Cancer Genome Atlas (TCGA). Besides, messenger RNA (mRNA) and protein expressions of the six-gene signature were explored using Oncomine, Human Protein Atlas (HPA) and the International Cancer Genome Consortium (ICGC).

**Results:** A total of 8,306 differentially expressed genes (DEGs) were obtained between HCC (*n* = 115) and normal tissues (*n* = 52). Top 5,000 significant genes were selected and subjected to the weighted correlation network analysis (WGCNA), which constructed nine gene co-expression modules that assign these genes to different modules by cluster dendrogram trees. By analyzing the most significant module (red module), six genes (SQSTM1, AHSA1, VNN2, SMG5, SRXN1, and GLS) were screened by univariate, LASSO, and multivariate Cox regression analysis. By a survival analysis with the HCC data in TCGA, we established a nomogram based on the six-gene signature and multiple clinicopathological features. The six-gene signature was then validated as an independent prognostic factor in independent HCC cohort from ICGC. Receiver operating characteristic (ROC) curve analysis confirmed the predictive capacity of the six-gene signature and nomogram. Besides, overexpression of the six genes at the mRNA and protein levels was validated using Oncomine and HPA, respectively.

**Conclusion:** The predictive six-gene signature and nomograms established in this study can assist clinicians in selecting personalized treatment for patients with HCC.

## Introduction

Hepatocellular carcinoma (HCC) is one of the most common malignancies worldwide. The mortality rate of HCC ranks second among all cancers, and HCC has a higher rate in developing countries compared to developed countries (El-Serag and Rudolph, 2007). Approximately 70% of HCC relapse within 5 years after receiving resection or ablation (Cancer Genome Atlas Research Network Electronic Address and Cancer Genome Atlas Research, 2017). The main causes leading to the poor prognosis are tumor metastasis and postoperative recurrence (Budhu et al., 2006). Abnormal expression of messenger RNAs (mRNAs) plays critical roles in a variety of biological processes. Recent studies have documented that mRNAs can function as potential biomarkers in cancer prognosis (Wang et al., 2018). Therefore, there is an urgent need in screening biomarkers of HCC to show therapeutic effects, reduce mortality, and improve the prognosis. A routine prognostic assessment tool for HCC patients was clinical pathological staging. However, HCC is always with clinical heterogeneity. For example, the clinical heterogeneity caused by the simultaneous presence of two life-threatening diseases, cancer and cirrhosis, often affects the effect of routine prognosis assessment. In order to provide more clinically beneficial treatment strategies for high-risk populations, there is an urgent need to develop a new prognostic prediction model as a supplement to the prediction outcomes of clinical staging.

During the last decades, gene sequencing and bioinformatic analysis have been widely used to screen genetic alterations at the genome level, which have helped us identify the differentially expressed genes (DEGs) and functional pathways involved in the progression of HCC. It was reported that epithelial cell adhesion molecule (Yamashita et al., 2008), CD24 (Woo et al., 2008), and TGF-beta (Coulouarn et al., 2008) were associated with the overall survival (OS) of HCC inpatients. However, false-positive rates in a single cohort analysis make it difficult to obtain reliable results. Thus, in the present study, we identify biomarkers of HCC by extracting a dataset of HCC patients from the Gene Expression Omnibus database (GEO). Then, we established a gene signature for HCC in Cancer Genome Atlas (TCGA) and established an integrated nomogram by combining multiple clinicopathological factors including the gene signature. Subsequently, the six-gene signature was verified in an independent external HCC cohort in International Union of Cancer Genome (ICGC). Besides, expression status of the six-gene signature in human HCC tissues at the mRNA and protein levels was explored using the Oncomine and the Human Protein Atlas (HPA) databases, respectively. In summary, we aim to establish a genetic marker and prognostic model that can predict the OS of HCC patients by bioinformatics methods. And this model could assist physicians to develop more individualized treatment plans.

## Materials and Methods

### Data Source

The mRNA expression profile of HCC patients used to identify differentially expressed genes was derived from GEO, which was calculated on the Illumina HiSeq RNA sequencing (RNA-seq) platform and contained 115 HCC tissues and 52 adjacent non-tumor tissues (ANTTs) as of August 13, 2018 (GSE76427). The training dataset with HCC mRNA expression profiles and clinical information used to construct multi-gene signature was obtained from TCGA. The validation dataset with mRNA expression profile and clinical information used to verify the multi-gene signature was downloaded from ICGC. The above three databases are publicly available and open-access, and the present study followed the data access policy and publishing guidelines of these databases. Therefore, no local ethics committee is required to approve this study.

### Identification of DEGs Between HCC and Non-Cancerous Tissues

Firstly, we obtained raw sequencing data for HCC mRNA including 41,718 mRNA expression profiles from the GEO database. Then, the DEG was calculated using the limma R package (Ritchie et al., 2015). DEGs with absolute log2 fold change (FC) > 1 and adjusted *P* value <0.05 were considered to be included for subsequent analysis.

### Co-Expression Gene Network Based On RNA-Seq Data

The weighted correlation network analysis (WGCNA) was used to construct the gene co-expression network (Langfelder and Horvath, 2008). Firstly, to construct a gene expression similarity matrix, we calculate the absolute value of the Pearson's correlation coefficient between gene *i* and gene *j*:

$$S_{ij}=|(1+\text{cor}(x_{i}+y_{j}))/2|,$$

where *i* and *j* represent the amount of expression of the *i* and *j* genes, respectively. Then, the gene expression similarity matrix was ​​ converted into an adjacency matrix, and the network type is signed. *β* is a soft threshold, which is actually the Pearson's correlation coefficient *β* of each pair of genes (Horvath et al., 2006). This step can strengthen strong correlation and weaken weak correlation from the index level:

$$a_{ij}={\left|(1+\text{cor}(x_{i}+y_{j}))/2\right|}^{\beta }.$$

The next step was to convert the adjacency matrix into a topological matrix. The topological overlap measure (TOM) was used to describe the degree of association between genes:

$$\text{TOM}=\left(\sum_{\mu \neq ij}\alpha_{i\mu }\alpha_{\mu j}+\alpha_{ij}\right)/(\min(\sum_{\mu }\alpha_{i\mu }+\sum_{\mu }\alpha_{j\mu })+1-\alpha_{ij}).$$

TOM indicates the degree of dissimilarity between gene *i* and gene *j*. We conducted hierarchical clustering of genes using 1-TOM as a distance, and then used the method of dynamic cut tree for module identification. The most representative gene in each module was called the eigenvector gene, referred to as ME, which represents the overall level of gene expression within the module:

$$\text{ME}=\text{princomp}(x_{ij}^{q}),$$

where *i* represents the gene in module *q* and *j* represents the chip sample in module *q*. We use Pearson's correlation between the expression profile of a gene in all samples and the ME expression profile of an eigenvector gene to measure the identity of the gene in the module. We called it module membership (MM):

$${\text{MM}}_{i}^{q}=\text{cor}(x_{i},{\text{ME}}^{q})$$

where ME represents the expression profile of the *i* gene.

### Functional Enrichment Analysis

Enrichment analysis of Gene Ontology (GO) and Kyoto Encyclopedia of Genes and Genomes (KEGG) pathway for genes in the most significant modules of the WGCNA analysis was performed using the clusterProfiler R package (Yu et al., 2012).

### Definition of the Gene-Related Prognostic Model

Univariate, the least absolute shrinkage and selection operator (LASSO), and multivariate Cox regression analyses were used to study the correlation between patient OS and gene expression levels (Tibshirani, 1997). Firstly, we used univariate Cox regression analysis to identify genes associated with OS, and then applied LASSO Cox regression to further narrow the range of HCC marker genes. After that, multiple Cox regression analysis was applied to assess whether marker genes could be an independent prognostic factor for patient survival. A multi-gene marker-based prognostic risk score was established based on a combination of regression coefficients from the multivariate Cox regression model (*β*) multiplied by their expression levels. Prognostic index (Pi) = (*β* * expression level of SQSTM1) + (*β* * expression level of AHSA1) + (*β* * expression level of VNN2) + (*β* * expression level of SMG5) + (*β* * expression level of SRXN1) + (*β* * expression level of GLS). Taking the median risk score as a cutoff value, 365 HCC patients from TCGA were divided into high- and low-risk groups. Kaplan–Meier (KM) survival curves and time-dependent receiver operational feature (ROC) curve analyses were made to assess the predictive capacity of the model. Decision curve analysis (DCA) curves were used to visually assess the clinical benefit of the model. Besides, the prognostic model was validated in an independent cohort from ICGC.

### Prognostic Model Based on Six-Gene Signature as an Independent Predictor for OS

We used univariate and multivariate Cox regression analysis to assess whether the prognostic model could be independent of other clinicopathological variables (including age, gender, tissue registration, pathological stage, T staging, and risk score) for HCC patients. Clinical features were selected as an independent variable, and OS was selected as the dependent variable to calculate the hazard ratio (HR) and the 95% confidence interval, two-sided *P* value.

### Validation of the Six-Gene Signature Using Multiple Databases

We used an online microarray database called Oncomine (http://www.oncomine.org) to analyze the mRNA expression of the gene signature between HCC tissues and normal liver tissues (Rhodes et al., 2004). The threshold settings were as follows: *P* value: 0.01; fold change: 2; gene rank: 10%. The datasets, sample size, fold change, *t* test, and *P* value were all derived from studies with statistical differences. In addition, immunohistochemical images were downloaded from publicly available human protein maps (http://www.proteinatlas.org) for comparison of protein expression levels related to the gene signature (Uhlen et al., 2010). We obtained an independent HCC cohort from ICGC, extracted the expression levels of six-gene signature, and compared the expression levels of six-gene signature between HCC and non-tumor tissues using Wilcoxon signed-rank test (two-sided *P* values, and *P* < 0.05 indicates significant statistical differences).

### Establishment and Evaluation of the Nomograms for HCC Survival Prediction

Nomogram is an effective method for predicting the prognosis of cancer patients by simplifying the complex statistical prediction model into a profile chart for assessing the probability of OS in individual patients (Park, 2018). In this study, we included all independent clinical pathological prognostic factors selected from Cox regression analysis to construct a nomogram which can assess the OS probability of 1, 3, and 5 years in HCC patients. The prediction probability of the nomogram was compared with the observed actual probability by the calibration curve to verify the accuracy of the nomogram. Overlapping the reference line indicates that the model is accurate. ROC analysis was used to compare the prediction accuracy between the nomogram of combined model and the nomogram for each single clinical pathological prognostic factor.

## Results

### Study Process and Summary of Patients' Information

**Figure 1:**
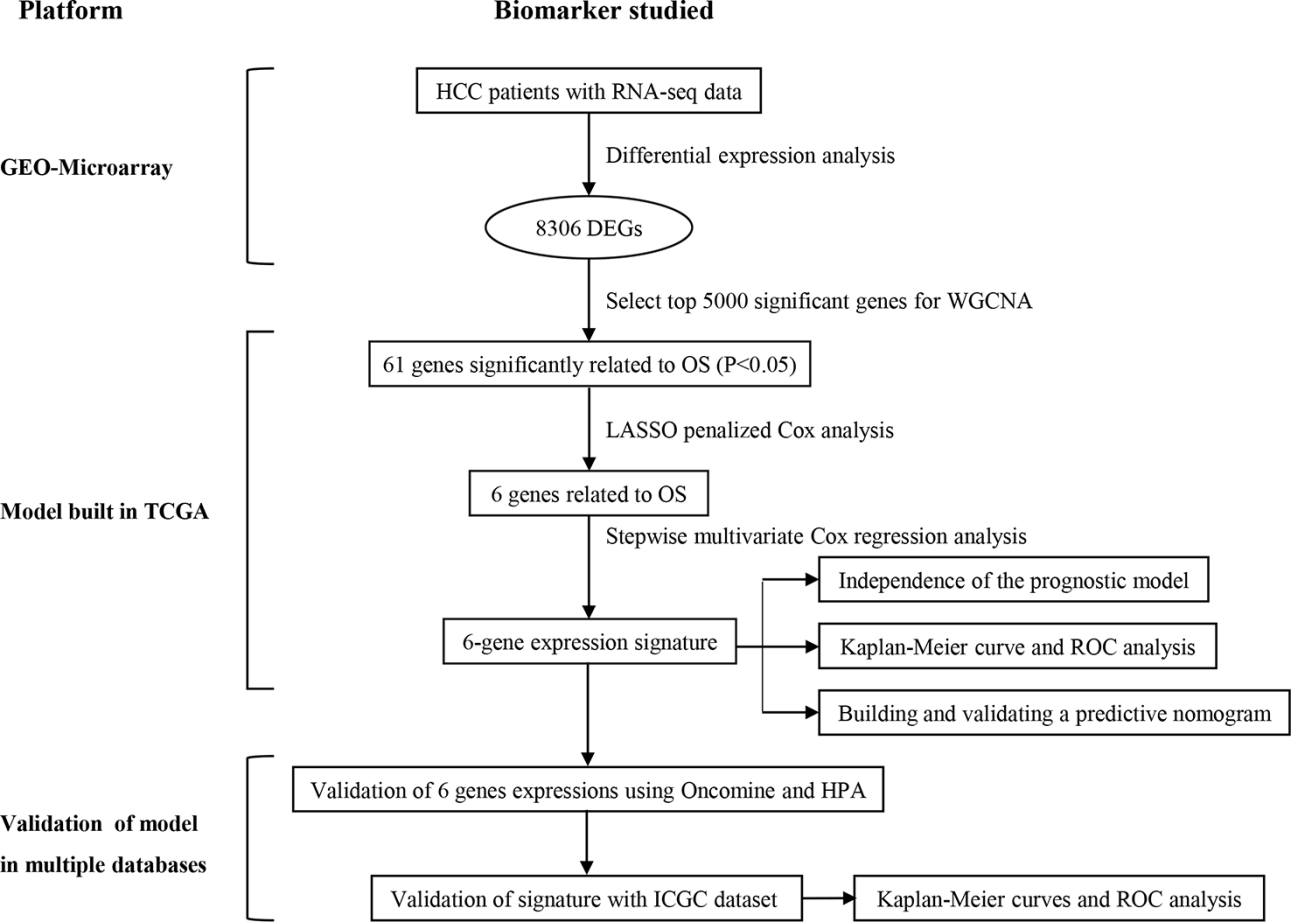
Overall flowchart of this study.

**Figure 1** is a flowchart for the entire work of this study. The detailed construction process of the OS prediction model for patients with HCC was shown in this chart. Patients' information in the GEO, TCGA, and ICGC cohorts was shown in **Table 1**.

**Table 1:** Patients' information in the GEO, TCGA, and ICGC cohorts.

Clinical characteristics		Total	%
GSE 76427 in GEO		115	100
Survival status	Survival	92	80
	Death	23	20
Age	≤65 years	65	56.5
	>65 years	50	43.5
Gender	Female	22	19.1
	Male	93	80.9
Stage	I	55	47.8
	II	35	30.4
	III	21	18.3
	IV	3	2.5
TCGA		365	100
Survival status	Survival	239	65.48
	Death	126	34.52
Age	≤65 years	227	62.19
	>65 years	138	37.81
Gender	Male	246	67.40
	Female	119	32.60
Histological grade	G1	55	15.07
	G2	175	47.95
	G3	118	32.33
	G4	12	3.29
Stage	I	170	46.56
	II	84	23.01
	III	83	22.74
	IV	4	1.10
T classification	T1	180	49.32
	T2	91	24.93
	T3	78	21.37
	T4	13	3.56
ICGC		232	100
Survival status	Survival	189	81.47
	Death	43	18.53
Age	≤65 years	90	38.79
	>65 years	142	61.21
Gender	Male	171	73.71
	Female	61	26.29
Stage	I	36	15.52
	II	106	45.69
	III	71	30.60
	IV	19	8.19
Prior malignancy	No	202	87.07
	yes	30	12.93

### Identification of DEGS with Prognosis Value in HCC

**Figure 2:**
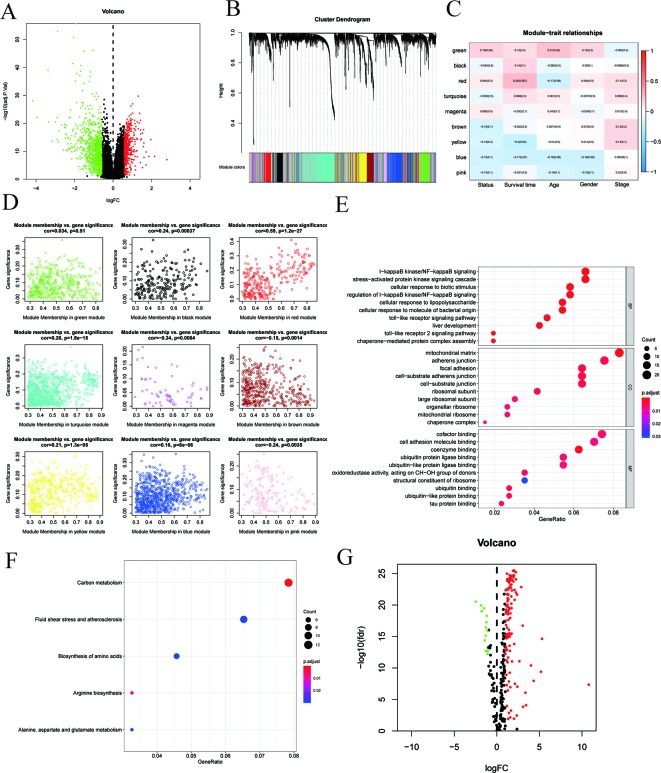
ldentification of prognostic genes in hepatocellular carcinoma patients. **(A)** Volcano plot showing differentially expressed genes (DEGs) in hepatocellular carcinoma samples. **(B)** Clustering dendrogram of genome-wide genes in hepatocellular carcinoma samples. **(C)** Correlation between modules and traits. Absolute values of correlation coefficients between hepatocellular carcinoma status and modules greater than 0.15 were considered as hepatocellular carcinoma-related modules. **(D)** Module membership in nine hepatocellular carcinoma-related modules. The *red* module was the most significant module. **(E**, **F)** GO and KEGG analysis revealed the most significant biological process (BP), molecular function (MF), cellular component (CC), and pathways correlated with the high-risk group genes in the red module. **(G)** Volcano plot revealed DEGs in the red module.

As shown in the volcano map (**Figure 2A**), a comparative analysis of mRNA expression profiles between HCC tissues (*n* = 115) and ANTTs (*n* = 52) identified 8,306 significantly differentially expressed mRNAs (logFC > 1 or logFC < −1, adjusted *P* < 0.05). Then, all DEGs were sorted in ascending order according to the adjusted *P* value, and the top 5,000 genes were selected and subjected to WGCNA, which constructed gene co-expression modules that assign these genes to different modules by cluster dendrogram trees (**Figure 2B**). Gene numbers of each module in WGCNA are shown in **Table 2**. The correlation coefficients between each co-expressed gene module and the clinical features of HCC are shown in **Figure 2C**, and the module membership vs. gene significance analysis of the nine HCC-related modules is shown in **Figure 2D**. **Figures 2C, D** show that the red module was not only with the largest correlation coefficient regarding to OS time (0.25) but also with the most significant module membership relevance to gene significance (module membership vs. gene significance: cor = 0.59, *P* = 1.2e−27). Thus, the red module was considered as the most important module related to the prognosis of HCC. And genes of the red module were extracted for GO and KEGG analysis. GO analysis that showed the most significant biological process (BP), molecular function (MF), and cellular component (CC) were I-kappa B kinase/NF-kappa B signaling, mitochondrial matrix, and cofactor binding, respectively (**Figure 2E**). And KEGG analysis showed the key pathways correlated with the HCC samples: carbon metabolism, fluid shear stress and atherosclerosis, biosynthesis of amino acids, arginine biosynthesis, and alanine–aspartate–glutamate metabolism (*P*_adjust_ < 0.05) (**Figure 2F**).

**Table 2:** Gene numbers of each module in WGCNA.

Module	Number
Black	216
Blue	792
Brown	449
Green	379
Gray	1,345
Magenta	63
Pink	146
Red	280
Turquoise	907
Yellow	423

### Constructing the Six-Gene Signature for Risk Scoring and Survival Prediction

**Figure 3:**
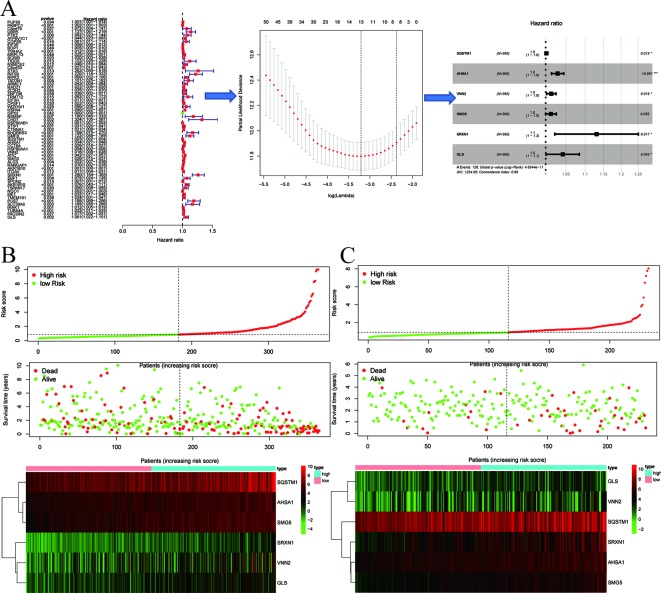
Signature-based risk score is a promising marker in the training and validation cohorts. **(A)** The process of building the signature containing six genes most correlated with overall survival (OS) in the training set. The hazard ratios (HRs), 95% confidence intervals (CIs) calculated by univariate Cox regression, and the coefficients calculated by multivariate Cox regression using LASSO are shown. **(B**, **C)** Risk score distribution, survival overview, and heatmap for patients in the TCGA **(B)** and ICGC **(C)** datasets assigned to high- and low-risk groups based on the risk score.

A differential gene expression analysis was conducted (**Figure 2G**), and 61 key genes were selected for further analysis in TCGA. The entire process of extracting stable genes from the 61 prognostic-related genes in the HCC dataset from the TCGA to build a survival prediction model is presented in **Figure 3A**. To build a clinical survival prognostic model for HCC, we used TCGA as a training dataset and applied the LASSO Cox regression analysis to identify stable markers from 61 survival-related candidates. By forcing the sum of the absolute values of regression coefficients to be less than a fixed value, some coefficients were reduced to zero, and then we used relative regression coefficients to identify the most stable prognostic markers and apply cross-validation to avoid overfitting of the LASSO Cox model. Parameters for building multivariate COX model are shown in **Table 3**, and six filter markers—SQSTM1, AHSA1, VNN2, SMG5, SRXN1, and GLS—are associated with high risk (HR > 1).

**Table 3:** Parameters for building multivariate COX model.

Gene	Co-ef	HR	HR.95%L	HR.95%H	*P* value
SQSTM1	0.001803	1.001805	1.000376	1.003236	0.013287
AHSA1	0.028803	1.029222	1.014274	1.044391	0.000114
VNN2	0.013683	1.013777	1.002275	1.025411	0.018759
SMG5	0.012785	1.012867	0.999704	1.026203	0.055423
SRXN1	0.122996	1.13088	1.022251	1.251051	0.016983
GLS	0.041246	1.042108	1.001332	1.084545	0.042835

Co-ef : co-efficient, HR: hazard ratio.

Then six genes were then applied to build a polygenic signature for prognostic prediction based on the minimum criteria. Subsequently, the risk score of each HCC patient from the training set was calculated using the coefficients obtained from the LASSO algorithm. To test the relationship between six identified genes and the prognosis of HCC patients, we constructed a prognostic model based on six-gene signature. Then, 365 HCC patients with follow-up information were divided into low-risk group and high-risk group according to the median value of risk scores among all HCC patients in the training set. Comparing the survival status and the six-gene expressions of the two groups, we found that the high-risk group was with poor prognosis and with higher expression of the six identified genes (**Figure 3B**).

Next, we proved our findings in the training set by validating the prognostic prediction function of the six-gene signature in an independent dataset from ICGC. We extracted microarray data from 243 HCC patients with follow-up information from the validation set and then calculated the risk score for each patient by using the same formula in the training set. Taking the median risk score as a cutoff value, the HCC patients in the validation set were divided into high- (*n* = 122) and low-risk (*n* = 121) groups, and the survival status and six-gene expressions were compared between the two groups. A similar result to the training set was obtained: the high-risk group was with poor prognosis and with higher six-gene expression level than the low-risk group (**Figure 3C**).

### Kaplan–Meier and Time-Dependent ROC Curves of Six-Gene Signature

**Figure 4:**
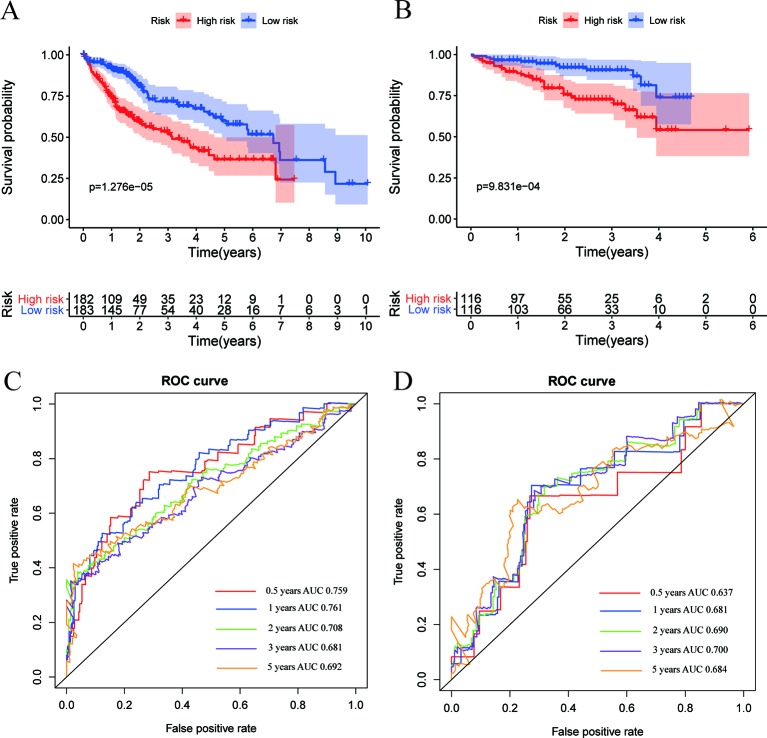
Expression and survival analysis in training and validation datasets. **(A**, **B)** Kaplan–Meier overall survival (OS) curves for patients in the TCGA **(A)** and ICGC **(B)** datasets assigned to high- and low-risk groups based on the risk score. Patients with a high risk score exhibited poorer OS in the training and validation cohorts. **(C**, **D)** ROC curves showed the predictive efficiency of the risk signature for patients in the TCGA **(C)** and ICGC **(D)** datasets on the survival rate.

The Kaplan–Meier survival curve was applied to present a comparison of the OS of the two groups divided by the median risk score. Besides, the area under the ROC curve (AUC) of the time-dependent ROC curve was used to assess the prognostic ability of the six-gene signature, and a higher AUC means the better the model performance. We found that there was a significant difference on OS between the high- and low- risk groups in the TCGA dataset (*P* < 0.0001) (**Figure 4A**). The AUCs of the six-gene signature corresponding to 0.5, 1, 2, 3, and 5 years of survival were 0.759, 0.761, 0.708, 0.681, and 0.692, respectively, suggesting that the prediction model had high sensitivity and specificity (**Figure 4C**). As shown in the other Kaplan–Meier curve (**Figure 4B**), the OS was significantly increased in the low-risk group compared to the high-risk group in the independent validation dataset from the ICGC dataset (*P* < 0.001). This result was consistent with our previous findings in the training cohort in TCGA dataset. As shown in **Figure 4D**, the AUCs of the six-gene signature model corresponding to 0.5, 1, 2, 3, and 5 years of survival were 0.637, 0.681, 0.690, 0.700, and 0.684, respectively, further confirming that the six-gene signature had high sensitivity and specificity and can be used as a reliable predictor of OS in HCC patients.

### Prognostic Risk Scores were an Independent Prognostic Factor from the Other Clinicopathological Features

**Figure 5:**
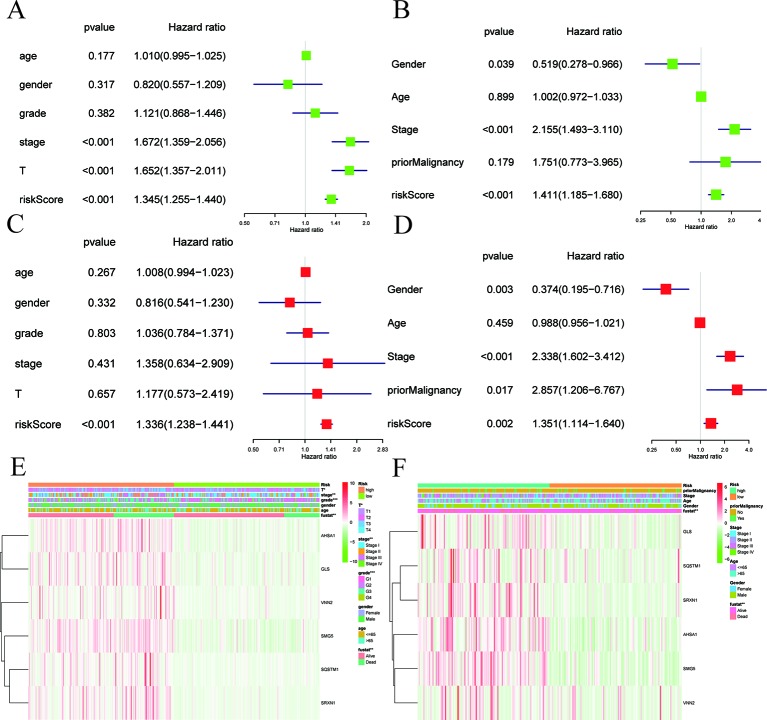
Cox regression analyses of the association between clinicopathological factors and OS. **(A**–**F)** Univariate/multivariate Cox regression analyses and heatmaps of the association between clinicopathological factors (including the risk score) and overall survival (OS) of patients in the TCGA **(A**, **C**, **E)** and ICGC **(B**, **D**, **F)** datasets.

As shown in **Figure 5**, the risk score can be used as an independent factor in predicting OS. Univariate and multivariate Cox regression analyses were applied to assess independent predictive values ​​ for the six-gene signature in HCC patients. In the TCGA dataset, univariate Cox regression suggested that risk scores, pathological staging, and T staging had a prognostic value, while age, gender, and histological grades were not associated with survival (**Figure 5A**). Then, multivariate Cox regression analysis suggested that only risk score was an independent prognostic factor associated with OS (**Figure 5C**). Next, we again used univariate and multivariate Cox regression analysis to validate whether the risk score can be used as an independent prognostic indicator in an independent HCC cohort from ICGC. Univariate Cox analysis suggested that risk score and pathological stage were associated with OS (*P* < 0.05; **Figure 5B**). Multivariate Cox regression analysis showed that risk scores, prior malignancy, and pathological stage were associated with OS (*P* < 0.05; **Figure 5D**). These results confirmed that risk scores based on six-gene signature can be used as an independent predictor of prognosis in HCC patients. Shown in the heat map are the expression levels of the six-gene signature in low-risk and high-risk HCC patients and the distribution of clinicopathological features between the low-risk and high-risk groups. It is suggested that there were significant differences in six-gene signature expression and OS between the high-risk group and the low-risk group both in the TCGA (**Figure 5E**) and ICGC (**Figure 5F**) datasets.

### Subgroup Analysis of OS Based on Multiple Classification Methods

**Figure 6:**
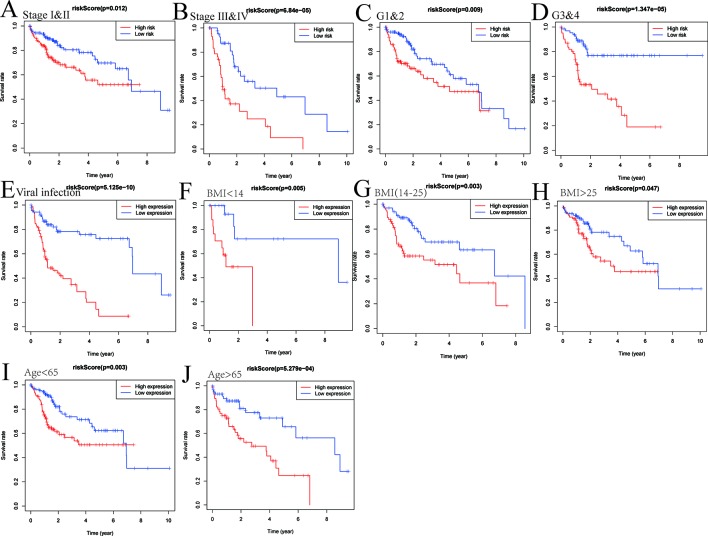
The six-gene-based risk score is a promising marker for overall survival (OS) in subgroups. Subgroup analysis of OS based on pathological staging **(A**, **B)**, grading **(C**, **D)**, viral hepatitis **(E)**, BMI **(F**–**H)**, and age **(I**, **J)** of hepatocellular carcinoma (HCC) patients.

As shown in **Figure 6**, the survival analysis was conducted after risk score grouping based on six-gene signature expressions. We explored the expression profiles of six genes in different TNM stages, histological grades, viral hepatitis infection, BMI, and age in TCGA. Risk score based on six-gene signature was proven to be a potential marker for predicting OS in different subgroups, including stages I–II of TNM (*P* = 0.012), stages III–IV (*P* < 0.0001), G1 and G2 (*P* = 0.009), G3 and G4 (*P* < 0.0001), viral infection (*P* < 0.0001), BMI < 14 (*P* = 0.005), BMI(14–25) (*P* = 0.003), BMI > 25 (*P* = 0.047), age < 65 (*P* = 0.003), and age > 65 (*P* < 0.001).

### Validation of the Six mRNA Expressions

**Figure 7:**
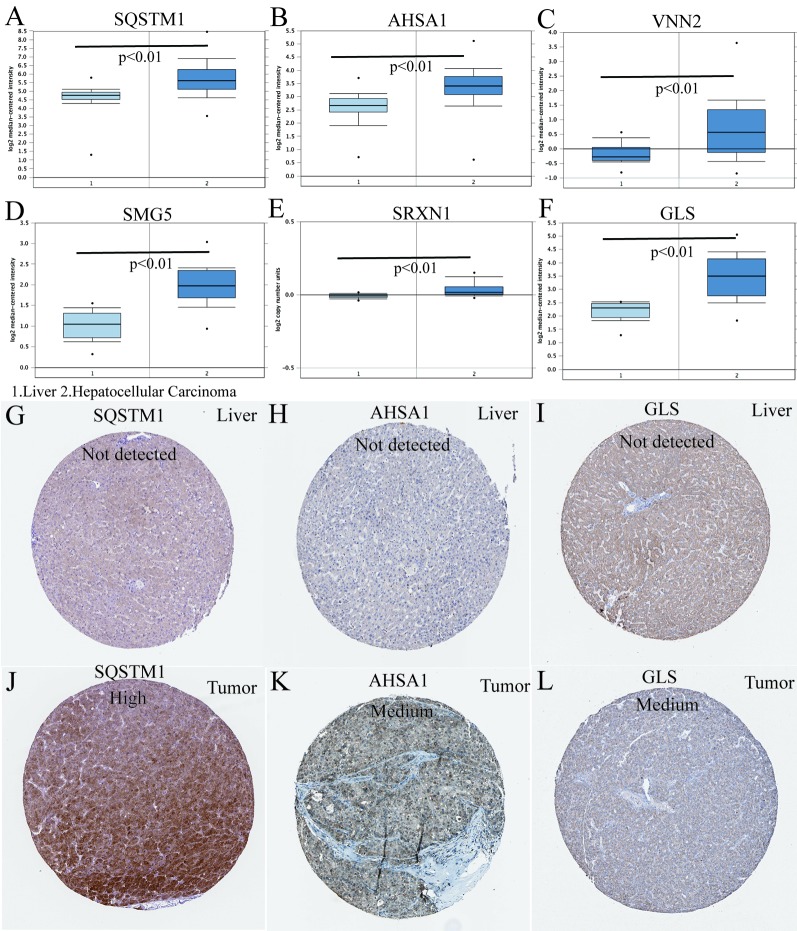
Differences in protein expression induced by six genes were verified in human tissue samples. **(A**–**F)** The mRNA expression levels of the six-gene signature in human cancers (conducted in Oncomine database). **(G**–**L)** Human Protein Atlas immunohistochemistry using anti-SQSTM1, anti-AHSA1, and anti-GLS antibodies. Normal liver **(G**–**I)** vs. tumor tissues **(J**–**L)**.

In the TCGA HCC cohort, all six genes were highly expressed in HCC compared to that in adjacent non-tumor liver tissues. Next, we aimed to further confirm the expression patterns of these six genes in HCC tissues in the Oncomine database. Consistent with our results in TCGA, the average expression levels of SQSTM1, AHSA1, VNN2, SMG5, SRXN1, and GLS in HCC tissues were significantly higher than those in normal liver tissues (**Figures 7A–F**). To determine the clinical relevance of the six genes' expression, we analyzed the expression of the proteins encoded by these six genes using clinical specimens from the HPA. Relative to its expression level in normal liver tissue, SQSTM1 was strongly positive, while AHSA1 and GLS were moderately positive in HCC tissues (**Figures 7G–L**). However, VNN2, SMG5, and SRXN1 were not found on the website.

### Building a Nomogram to Predict OS in HCC Patients

**Figure 8:**
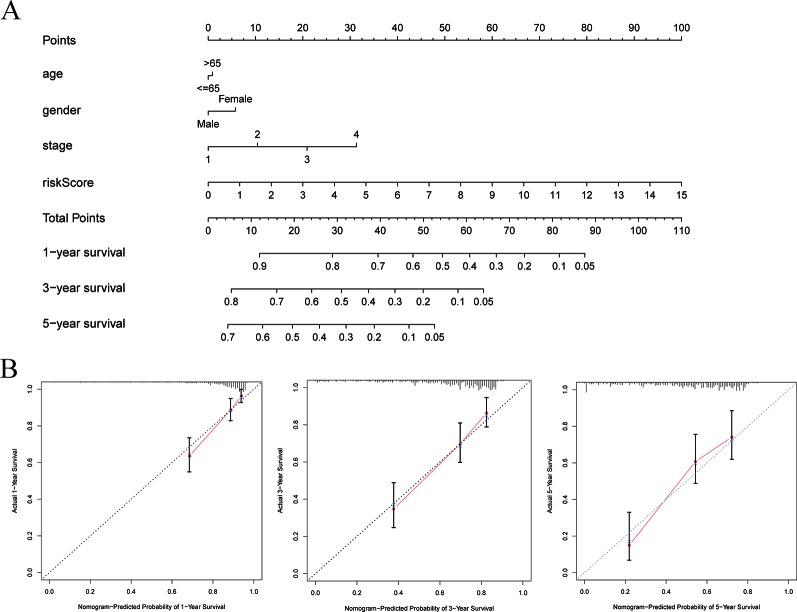
Construction of a nomogram for survival prediction. **(A)** Nomogram combining signature with clinicopathological features. **(B)** Calibration plot showing that nomogram-predicted survival probabilities corresponded closely to the actual observed proportions.

To establish a clinically applicable method for predicting the survival probability of patients with HCC, we developed a nomogram to predict the probability of the 1-, 3-, and 5-year OS in the TCGA cohort. The predictors of the nomogram included four independent prognostic factors (age, gender, pathologic stage, and six-gene signature). Subsequently, we constructed a nomogram that integrates clinical pathology features with six-gene signature to predict survival probabilities in HCC patients (**Figure 8A**). By calibration curve analysis, we found that the 1-, 3-, and 5-year survival probabilities predicted by the nomogram were closely related to the observed survival probability, which confirmed the reliability of the nomogram (**Figure 8B**).

### Assessing the Accuracy of the Nomograms by ROC Curves

**Figure 9:**
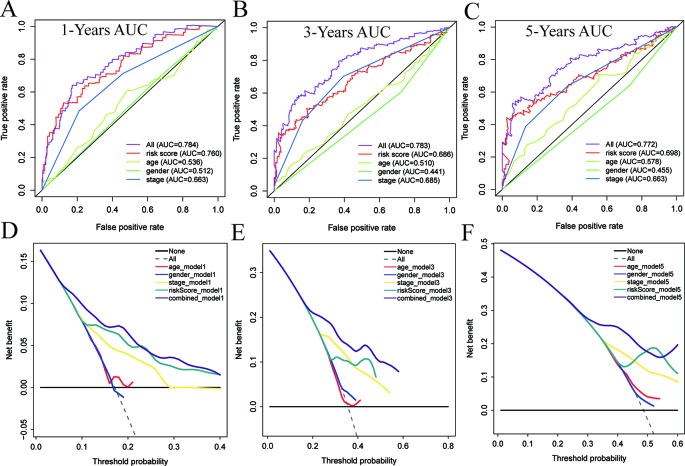
The time-dependent receiver operating characteristic (ROC) and decision curve analysis (DCA) curves of the nomograms. Time-dependent ROC curve analysis evaluates the accuracy of the nomograms **(A**–**C)**. The *purple*, *red*, *yellow*, *green*, or *blue solid line* represents the nomogram. The DCA curves can intuitively evaluate the clinical benefit of the nomograms and the scope of application of the nomograms to obtain clinical benefits **(D**–**F)**. The net benefits (*Y*-axis) as calculated are plotted against the threshold probabilities of patients having 1, 3-, and 5-year survival on the *X*-axis. The *gray dotted line* represents the assumption that all patients have 1-, 3-, and 5-year survival. The *black solid line* represents the assumption that no patients have 1-, 3-, or 5-year survival. The *red*, *blue*, *yellow*, *green*, or *purple solid line* represents the nomograms.

Time-dependent ROC curve analysis was used to evaluate the prediction accuracy of the integrated nomogram. The solid red line represents the integrated nomogram. In **Figures 9A–C**, the AUC of the integrated nomogram is the largest. Besides, all of AUCs of the integrated nomogram in **Figure 9** were above 0.77, suggesting that nomograms constructed by integrated factors are the best way to predict survival in HCC patients both for short-term and long-term survival compared to models constructed by a single prognostic factor. However, we also found that integrated predictions of the 3- and 5-year AUC of the integrated model are lower than that of 1 year, suggesting that the short-term prediction ability of the nomogram may be stronger than the long-term prediction ability. Besides, as shown in **Figures 9D–F**, the net benefits as calculated are plotted against the threshold probabilities of patients having 1-, 3-, and 5-year survival, and the results suggest that the net benefits of the integrated model were better than other models.

## Discussion

Due to the complex molecular mechanisms, HCC remains one of the most life-threatening malignancies in the world. Therefore, prognostic biomarkers are urgently needed to predict the outcome and to outline an individualized treatment plan for HCC patients. With the development of gene sequencing technology, some potential gene markers with predictive value for HCC patients have been identified. However, the number of such markers is still limited. In order to improve the prognosis of HCC, it is urgent to screen out more biomarkers with higher prediction accuracy in predicting prognosis.

In the present study, we identified potential gene biomarkers by analyzing the gene expression profiles of a HCC cohort in GEO. The DEGs between HCC samples and ANTTs were identified. Then, univariate, LASSO, and multivariate Cox analysis were used to further narrow the marker range and establish a risk model for predicting HCC prognosis. Our study found that high expression levels of six genes, including SQSTM1, AHSA1, VNN2, SMG5, SRXN1, and GLS, were associated with poor prognosis in HCC patients. We evaluated the model performance using the ROC curve of the six-gene signature. The results showed that the AUCs of the ROC curves for 0.5-, 1-, 2-, 3-, and 5-year survival prediction models were 0.637, 0.681, 0.690, 0.700, and 0.684, respectively, suggesting the six-gene signature was with good survival prediction performance. Then, we not only demonstrated that the six-gene signature was an independent prognostic factor for HCC patients superior to traditional clinicopathological factors but also verified their survival prediction ability in an external HCC cohort in ICGC. Thus, we believe that dividing HCC patients into high-risk group and low-risk group by the six-gene-based risk scoring model can be used for early prevention or detection of HCC recurrence in high-risk population.

Nomograms are a tool commonly used for tumor disease assessment to provide probabilistic predictions for individual patients. In our study, we constructed a nomogram that can predict the OS in HCC patients. The calibration curve indicates that the survival rate predicted by the nomogram is basically consistent with the actual observed survival rate in the dataset, indicating that the nomogram had good predictive performance. At the same time, we also proved that the use of the nomogram constructed by the combined model has better predictive performance than the nomogram constructed by a single HCC risk factor.

There were six genes identified for constructing the predictive model in this study. SQSTM1 is primarily involved in TNF signaling and the innate immune system. AHSA1 is primarily involved in ATPase activator activity. VNN2 is primarily involved in hydrolase activity. SMG5 is primarily involved in protein phosphatase 2A binding. And SRXN1 and GLS are involved in oxidoreductase activity and glutaminase activity, respectively. Combined with the results of GO and KEGG analysis, these perceptions suggested that abnormalities in energy metabolism and amino acid metabolism may play an important role in HCC.

HCC is a heterogeneous tumor that occurs through multiple pathway activations and molecular changes. Therefore, molecular heterogeneity affects the efficacy of prognostic evaluation by a single molecular marker. At the same time, some studies found that low survival rates of HCC were associated with strong cell proliferation and anti-apoptotic gene expression. These processes often involved multiple genes. And compared with single gene markers, multi-gene markers were always with more accurate prediction capacity for HCC (Lee et al., 2004). Bioinformatics methods were usually used to establish multi-gene signature for predicting the prognosis of HCC (Ye et al., 2003), and multi-gene signature is usually established by strategies including training, testing, and independent cross-validation (Roessler et al., 2010). Prediction capacity of a gene signature was significantly improved by the above strategies. It was reported that multi-gene signatures had a good predictive effect on venous metastasis (Budhu et al., 2006), progression (Roessler et al., 2012), recurrence (Kurokawa et al., 2004; Ho et al., 2006), and survival (Hoshida et al., 2013; Lim et al., 2013; Villa et al., 2016) for HCC. Initial multi-gene signatures often involved a large number of genes, and it affected the clinical application of the signature. It is believed that more user-friendly risk score models with a limited number of genes should be established to predict the prognosis of HCC patients (Kim et al., 2012). Recent study found a five-gene-based signature for HCC including HN1, RAN, AMP3, KRT19, and TAF9 (Nault et al., 2013). Now, it is believed that a combination model based on clinical, pathological, and gene signature will be more practical (Villanueva et al., 2011). At the same time, microRNAs (miRNAs) such as miR-517a (Toffanin et al., 2011), miR-125b (Li et al., 2008), and miR-26 (Ji et al., 2009) have been found to be associated with prognosis of HCC. There are also multiple gene markers based on multiple miRNAs and lncRNA (Budhu et al., 2008; Jiang et al., 2008).

In recent years, the identification of prognostic gene signature for HCC has been noted in many studies. For example, an eight-gene signature with a 5-year survival prediction AUC of 0.770 containing eight protein-coding genes (DCAF13, FAM163A, GPR18, LRP10, PVRIG, S100A9, SGCB, and TNNI3K) was established (Qiao et al., 2019). Subsequently, a six-gene signature (CSE1L, CSTB, MTHFR, DAGLA, MMP10, and GYS2) with a 5-year survival prediction AUC of 0.718 was established (Liu et al., 2019). In this study, we established a prognostic model with higher 5-year survival prediction AUC (0.772) based on a novel six-gene signature and further improved the predictive power of the HCC survival prediction model. To our knowledge, survival prediction models based on this six-gene signature have not been reported yet. Compared with traditional pathological staging and tissue grading, multi-gene signature of HCC has the advantages of higher prediction accuracy, more individualized test results, and reasonable sequencing costs. Therefore, six-gene signature has good prospects in clinical practice. In our study, we constructed and verified this six-gene signature by three independent datasets. More reasonable use of the biometric methods and mutual verification of multiple independent datasets make our study have more reliable results.

However, there were some limitations in this study. For example, the racial factors associated with sequencing samples and some potential prognostic factors may be not included in the model limited the predictive power of this model. In the future, we plan to use more rational bioinformatics strategies to improve the model. In summary, our results suggest that the six-gene-based prognosis model is a reliable tool for predicting OS in patients with HCC, and the nomogram containing six-gene signature can help to develop personalized HCC treatments in clinical practice. The challenge in the future is how to apply various genes signature reasonably in a particular stage of HCC.

## Data Availability Statement

The datasets analyzed in the current study are available in the TCGA repository (http://cancergenome.nih.gov/), the ICGC (https://icgc.org/), and GEO (https://www. ncbi.nlm.nih.gov/geo/).

## Ethics Statement

The usage of NIH controlled-access datasets was approved by the NCBI dbGaP.

## Author Contributions

JLiu designed and supervised the study and was a major contributor in editing the manuscript. WL and JLu analyzed and interpreted the data and were major contributors in writing the manuscript. ZM and JZ performed analysis and contributed to writing the manuscript. All authors read and approved the final manuscript.

## Conflict of Interest

The authors declare that the research was conducted in the absence of any commercial or financial relationships that could be construed as a potential conflict of interest.
